# New Appliances and Things Medical

**Published:** 1895-06-22

**Authors:** 


### NEW APPLIANCES AND THINGS MEDICAL.

[We shall be glad to receive, at our Office, 428, Strand, London, W.C., from the manufacturers, specimens of all new preparations and appliances which may be brought out from time to time.]

### OBERBRUNNEN MINERAL WATER.

(Ingram and Royle, 52, Farringdon Street, E.C.)

This Silesian mineral water has been renowned since 1601 for its salutary excellence, and has for many years been found specially efficacious in all affections of the organs of respiration, of the stomach, kidneys and bladder, in gout, scrofula, hæmorrhoids, and diabetes. Now that the practice of bottling the natural mineral waters is so common it is highly desirable that continental waters of repute should be tried in this country, although the majority of experts are agreed that the so-called cures which have been wrought by the taking of many of the mineral waters have been due in a great measure to the change of environment which the patient undergoes, which, accompanied by change of diet and freedom from work and anxiety, plays as an important a part as the daily visit to the pump-room in bringing about a restoration to health. The "Oberbrunnen" water is, however, remarkable for its marked alkaline character and for the large amount of bicarbonates of soda, lithia, and magnesia it contains. Our analysis shows that the bottled water contains no less than 331·5 parts of alkaline matter per 100,000 calculated as bicarbonate of soda. The total solid residue found on evaporating the water, and thereby converting the bicarbonates into neutral salts, was 315·5 parts per 100,000. In this residue our analyst found a small amount of iron and 39·4 parts of sulphate of soda per 100,000. We can confidently recommend this mineral water as being a genuine natural alkaline sulphated water.

